# Odour-Mediated Orientation of Beetles Is Influenced by Age, Sex and Morph

**DOI:** 10.1371/journal.pone.0049071

**Published:** 2012-11-07

**Authors:** Sarah E. J. Arnold, Philip C. Stevenson, Steven R. Belmain

**Affiliations:** 1 Natural Resources Institute, University of Greenwich, Chatham Maritime, Kent, United Kingdom; 2 Royal Botanic Gardens, Kew, Richmond, Surrey, United Kingdom; AgroParisTech, France

## Abstract

The behaviour of insects is dictated by a combination of factors and may vary considerably between individuals, but small insects are often considered en masse and thus these differences can be overlooked. For example, the cowpea bruchid *Callosobruchus maculatus* F. exists naturally in two adult forms: the active (flight) form for dispersal, and the inactive (flightless), more fecund but shorter-lived form. Given that these morphs show dissimilar biology, it is possible that they differ in odour-mediated orientation and yet studies of this species frequently neglect to distinguish morph type, or are carried out only on the inactive morph. Along with sex and age of individual, adult morph could be an important variable determining the biology of this and similar species, informing studies on evolution, ecology and pest management. We used an olfactometer with motion-tracking to investigate whether the olfactory behaviour and orientation of *C. maculatus* towards infested and uninfested cowpeas and a plant-derived repellent compound, methyl salicylate, differed between morphs or sexes. We found significant differences between the behaviour of male and female beetles and beetles of different ages, as well as interactive effects of sex, morph and age, in response to both host and repellent odours. This study demonstrates that behavioural experiments on insects should control for sex and age, while also considering differences between adult morphs where present in insect species. This finding has broad implications for fundamental entomological research, particularly when exploring the relationships between physiology, behaviour and evolutionary biology, and the application of crop protection strategies.

## Introduction

Chemical ecology is used increasingly in crop protection and vector control programmes, and exploiting the orientation behaviour of insects seeking food, mates or oviposition sites has provided environmentally sustainable solutions for some of mankind's major pest problems [1], [2]. Pest management can be implemented using effective applications of toxic synthetic pesticides [3]; however, for many farmers in developing countries the costs are prohibitive and the insecticidal products themselves may be adulterated [4]. The use of deterrent plant materials locally harvested from the wild presents a compelling alternative [5], [6]. The study of plant species to validate their efficacy or optimise their application, however, could be improved by understanding how different adult morphological types respond to different plants, as well as how age and sex influence odour mediated behaviour. In spite of these possible variables, many studies of insect odour-mediated behaviour do not explore potential effects of life-history traits such as sex [7]–[9] or morph [7], [10], [11]. Other studies examine only one sex, providing no information as to whether findings can be generalised to the other sex [10], [12]. In cases where sex, age or morph has been taken into account in insect olfactory behaviour, key differences in these behaviours have often emerged [13]–[15]. The evolution of behaviours such as host orientation and selection will depend on physiological constraints and life histories, and potential divergent behaviour within a species will have implications for understanding evolutionary biology and mechanics [16]–[18].

*Callosobruchus maculatus* F., the cowpea weevil, has two adult morphs, functionally similar to alate and apterous aphids. Flight polymorphisms are found in various insects clades, including pyrrhocorid bugs and some water striders and crickets [19], and can be mediated by physiological differences such as presence/absence of wings, length of wings and atrophy of flight muscles, or by the preferred behaviour of individuals. In *C. maculatus* conditions consisting of low larval densities, plentiful food and high moisture content, intermediate photoperiods and lower temperatures, cause the inactive form to develop [20], [21]. This is darker in colour, has a shorter life-span and does not fly, but is more fecund, with the females laying around 50 eggs. As the principle egg-layer, for oviposition deterrence or use of toxic plants, this morph is the primary target. At higher larval densities (correlated with increased temperature and bean moisture [22]) or in the presence of stressors such as low moisture or food availability, or extreme photoperiod, larval *C. maculatus* develop into active morph adults for dispersal [20]. Active morph production does not always increase linearly in response to larval crowding, and different strains have different genetic predispositions for active form production [21], [23]. Active adults are paler, and are less fecund, but live longer and fly readily [21], [24]. Females of this morph appear to remain in reproductive diapause in the absence of host material [22], [25]; this may have a bearing on their response to host odours. Fewer than half of active-form females lay any eggs at all in many laboratory cultures [21], [25] and those that do may lay as few as seven [24]. Even active individuals from strains recently obtained from the wild, which exhibit more reproductive activity, are still less fecund than the inactive morph [21]. The active morph may consequently represent less of a control target in established populations but individuals could be key pioneers able to start new populations, so control of these individuals is an important preventative strategy.

With such different activity stages in the life cycle, behavioural responses to odour cues may not be uniform. This has particular implications for management of stored product pests which employ technologies based on odour repellence. This is especially true in bruchid species such as *C. maculatus* where adults do not feed and oviposition material is thus of no nutritional value to them. Mated females might seek cowpea on which to oviposit; it is of value to males only if it also contains females. Equally, when confronted with volatile repellents one may observe differential responses in males and females, active and inactive morphs or older and younger individuals, with profound implications for pest management. Some target individuals (e.g. males, or the active morph) may be successfully repelled but if other individuals (e.g. females, or the inactive morph) are less strongly repelled or not affected at all, damage to stores and infestation of products is still a serious risk. Differences in the biology and life-history of males and females of this species (such as emergence weight and longevity) have already been observed [26], as well as their phenotypic responses to changing conditions [27], and so intraspecific variation at the behavioural level would also seem likely and should be quantified.

In this study we investigated differences in the response of *C. maculatus* adults to uninfested and infested host material and to the plant-derived repellent compound methyl salicylate [28], focusing on how these differences were associated with sex, age, morph and the level of infestation (ranging from an early infestation of eggs only to a heavy infestation with significant damage to beans). Using an olfactometer assay and multivariate analyses, we showed that these factors interact in determining behavioural responses.

## Methods

### (a) Insect culture

*C. maculatus* were cultured in laboratory conditions as described in Kestenholz et al. [29]. Adults were sexed according to shape of abdomen and markings on elytra [30] (females have a more oval-shaped abdomen with contrasting “eye” marks on the elytra and two dark stripes on the tip of the abdomen, whilst males have a rounder abdomen and more consistently patterned elytra, with a consistently pale abdominal tip); active/inactive form was determined both by the presence of flight activity, elytra size, and the intensity of pigmentation on elytra [31].

### (b) Olfactometry

**Figure 1:**
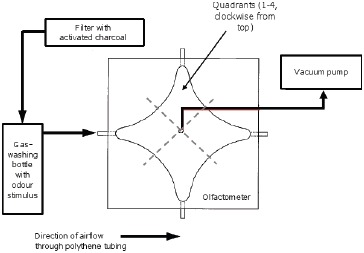
Olfactometer, showing the stimulus administration via airflow through one of the four arms. The pump draws air from the central chamber, and thus through each of the arms with an odour stimulus attached. Flow rate was checked regularly, ensuring that it was 200 ml/min through each arm (800 ml/min through the central hole).

A four-arm olfactometer as described in Jayasekara et al. [28] was used for all experiments. This consisted of a diamond-shaped, curve-edged arena (internal diameter 24.8 cm, apex to apex, total height 2.1 cm) made from an aluminium base-plate and sides and a glass lid, with four corners; each corner had a tube through which air can be drawn by pumping air from the arena through a central tube. The corner tubes were connected using Tygon tubing (internal Ø 6.4 mm) to four separate gas-washing bottles [32] (Fig. 1), each of which could be left empty or filled with an olfactory stimulus (e.g. uninfested or infested dried cowpeas). This ensured that, although the insect could detect the odour of the beans, it could neither see nor contact them. Air was drawn from the arena by a pump (Fisher Scientific UK Ltd., Loughborough, UK) at a rate of 800 ml/min, resulting in air being passed through each of the four arms at 200 ml/min; this was regulated with a flow-meter. All airstreams were passed through charcoal filters (Agilent Technologies, Santa Clara, USA) to remove impurities.

Individual insects were introduced to the arena singly. After every trial, the white paper floor of the arena was replaced. Paper provides additional traction for walking insects, and improves contrast between the insect and the background for motion tracking but also adsorbs chemicals that might be released by a previous test animal, with implications for behaviour of subsequent insects if the paper is not replaced. The arena was cleaned regularly with 70% ethanol to minimise odour residues. The arms of the olfactometer used for test and control stimuli were pseudo-randomised with each bout to control for any innate preferences for particular parts of the arena.

### (c) Orientation towards host odours

To examine the life-history parameters affecting orientation towards host (cowpea) odours a total of 84 insects were tested, with the breakdown of males and females, active and inactive morphs shown in Table 1. Each insect was tested only once, and prior to testing they had been kept in mixed-sex containers, so were assumed to be mated.

**Table 1:** Breakdown of individuals used in the host odours orientation experiment.

Sex	Morph	Number
Female	Active	21^1^
Female	Inactive	23
Male	Active	16
Male	Inactive	24^2^

1 One individual excluded as did not visit either host quadrant.

2 Four individuals excluded as did not visit either host quadrant.

The odour stimuli were uninfested cowpea in one arm and a second arm containing cowpea with varying levels of infestation. To create cowpea with early infestation, 50 g of beans were placed in a petri dish with five mated females less than 5 days old, and then left to oviposit for either 6 or 24 h. The beans were then used as a stimulus within 3 days of egg-laying. Heavily infested cowpea had had at least two generations of *C. maculatus* develop on the substrate and the beans had sustained heavy damage, containing a mixture of developmental larval stages. Previous studies have shown that the related species *Callosobruchus chinensis* responds differentially to cowpea containing different infestation levels [16], so it was considered necessary to explore more than one level of infestation of the host material in our study with the closely related *C*. *maculatus*.

Five beetles did not spend any of the recording period in either the infested or uninfested arms of the olfactometer and were consequently excluded from the statistical analysis.

### (d) Orientation in the presence of a repellent, botanically-derived compound: methyl salicylate

A total of 91 insects were tested in this experiment to investigate responses to the repellent compound, methyl salicylate, at concentrations of 0.1 mg/ml or 1 mg/ml. Five insects were excluded as they spent all their time in the control arms and, therefore, a preference between methyl salicylate and cowpea could not be determined. A full breakdown of the insects tested, by sex, morph and concentration of methyl salicylate used, is given in Table 2. Methyl salicylate is produced by a variety of species across the plant kingdom [33]–[35] and is the major volatile component in roots of *Securidaca longepedunculata* [36] which shows repellent effects to bruchids and cereal pests such as *Sitophilus zeamais* and *Prostephanus truncatus* [28].

**Table 2:** Breakdown of individuals tested in the methyl salicylate repellency experiment.

Concentration (mg/ml)	Sex	Morph	Number
0.1	Female	Active	16
0.1	Female	Inactive	17^1^
0.1	Male	Active	13^2^
0.1	Male	Inactive	15^3^
1.0	Female	Active	8
1.0	Female	Inactive	8
1.0	Male	Active	7
1.0	Male	Inactive	7^3^

1 One individual excluded as did not visit either host or methyl salicylate quadrant.

2 Two individuals excluded as did not visit either host or methyl salicylate quadrant.

3 One individual excluded as did not visit either host or methyl salicylate quadrant.

The odour stimuli in this experiment were 50 g uninfested cowpea, as before, and 20 µl of either 0.1 mg/ml or 1 mg/ml methyl salicylate (98% purity; Sigma-Aldrich, St Louis, MO, USA) in methanol, applied to a 30 mm Whatman type 1 filter paper disc.

### (e) Motion-tracking

In order to monitor insect behaviour continuously, rather than relying on manual records at set time-points, motion-tracking software was used to monitor the insects' movement during the recording period. This also circumvented the operator errors that may occur in single-moment event recording.

Each insect was monitored in the arena for 10 minutes using a digital camera above the arena recording to Ethovision 3.1 (Noldus Information Technology Ltd., Netherlands) [37] at a rate of 2.778 frames/second. The tracking software used a background subtraction method to detect the beetle (insects were darker than the background reference image). The insect's movements were tracked throughout the trial within the predefined arena, measuring the time spent in four designated quadrants of the arena, which permitted calculation of the percentage of time each insect chose to spend in the test and control odour streams.

### (f) Data analysis

Infestation level of cowpea, insect age, sex and morph were included in a multivariate Generalised Linear Model analysis with the amount of time spent in the positive control (uninfested cowpea) arm and the test (infested) arm used as dependent variables. This allowed preferences for infested and uninfested cowpea to be determined among insects according to age, sex, morph and the level of infestation of the cowpea. The data were analysed in the statistical package R [38], using a generalised linear model (GLM) with quasibinomial errors and a logit link. Initially, the model took into account all variables and interactive effects, which were then analysed for significance using an ANOVA with F-tests. Similar GLM analyses with ANOVA were also performed on the sets of methyl salicylate experimental results.

The effects and interactions which were significant (p<0.05) or marginal (p<0.1) were then entered into a refined generalised linear model with only those terms and re-analysed with ANOVA. The residuals were checked for normality, and indicated a good fit from the model. This analysis provided a more specific test of which contributing effects were most important in predicting the preference for infested over uninfested cowpea in *C. maculatus*. In addition, we ran a simpler analysis looking at the combined preferences for any (infested or uninfested) host material to see whether these differed between morph and sex of the beetles.

## Results

### (a) Host orientation

**Figure 2:**
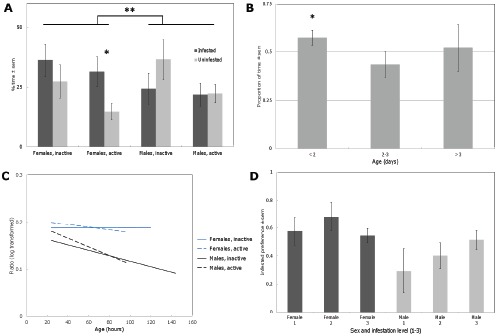
Preference (±s.e.m.) for infested over uninfested cowpea in *C. maculatus* adults, according to various characteristics. a) flight form and sex; b) preference expressed as a ratio, proportion of time spent in infested cowpea quadrant over proportion of time spent in both cowpea containing quadrants, by age alone; c) log-transformed data showing interactions between age, sex and flight form on preferences for infested and uninfested cowpea (expressed as ratio as above); d) interactive effects of sex and infestation level (from 1 = early infestation, 6 hours of egg-laying by 5 females, to 3 = very heavy, with multiple instars and emerging adults present). In b) and c), 0.5 indicates no preference between infested and uninfested cowpea. Dots and stars indicate a difference between bars or groups with a significance threshold of: **#**  =  0.1, *  =  0.05, **  =  0.01.

When the data were analysed without distinguishing between morph, sex or age of insect, beetles showed an overall slight preference for infested compared to uninfested cowpea (spending 27.3% versus 24.0% of their time in the infested and uninfested quadrants respectively). However, preferences were more diverse when distinguishing between males and females, active and inactive forms, age or the degree of infestation. Graphs showing breakdowns of behaviour by different traits are provided in Figure 2.

This generalised linear model revealed that preference for infested versus uninfested cowpea was controlled by multiple factors, specifically age (*p*<0.0001) and sex (*p* = 0.007) in isolation. Females prefer infested cowpea; at all ages, beetles showed large inter-individual variation in preference, but older beetles tend to show less preference for infested cowpea. There were also significant interactive effects of age and sex (*p* = 0.003), and age and morph (*p* = 0.03). This can be observed in Figure 2C: the males' preferences for infested cowpea declined further with age, whereas the females' did not show the same level of change.

When considering attraction to the odour of cowpea in general, independently of infestation, inactive-form individuals were overall more strongly attracted to the two bean quadrants considered together than active-form individuals (60.8±5.2% spent in the quadrants with cowpea odour, versus 45.4±4.3% for active individuals; t-test, *t* = 2.23, *p* = 0.029). The highest level of attraction was in inactive females (63.5±6.8%) and the lowest was in inactive males (44.2±5.7%).

These results indicate significant differences in the odour-mediated preferences of the inactive and active forms of *C. maculatus* when orienting towards host material, something which has not been previously reported. There are also important effects of sex in determining behaviour, specifically for the preference for infested versus uninfested material, and effects of age, with the youngest beetles tending to prefer infested over uninfested cowpea, intermediate aged beetles favouring uninfested material and the oldest beetles losing the preference.

### (b) Response to methyl salicylate

**Figure 3:**
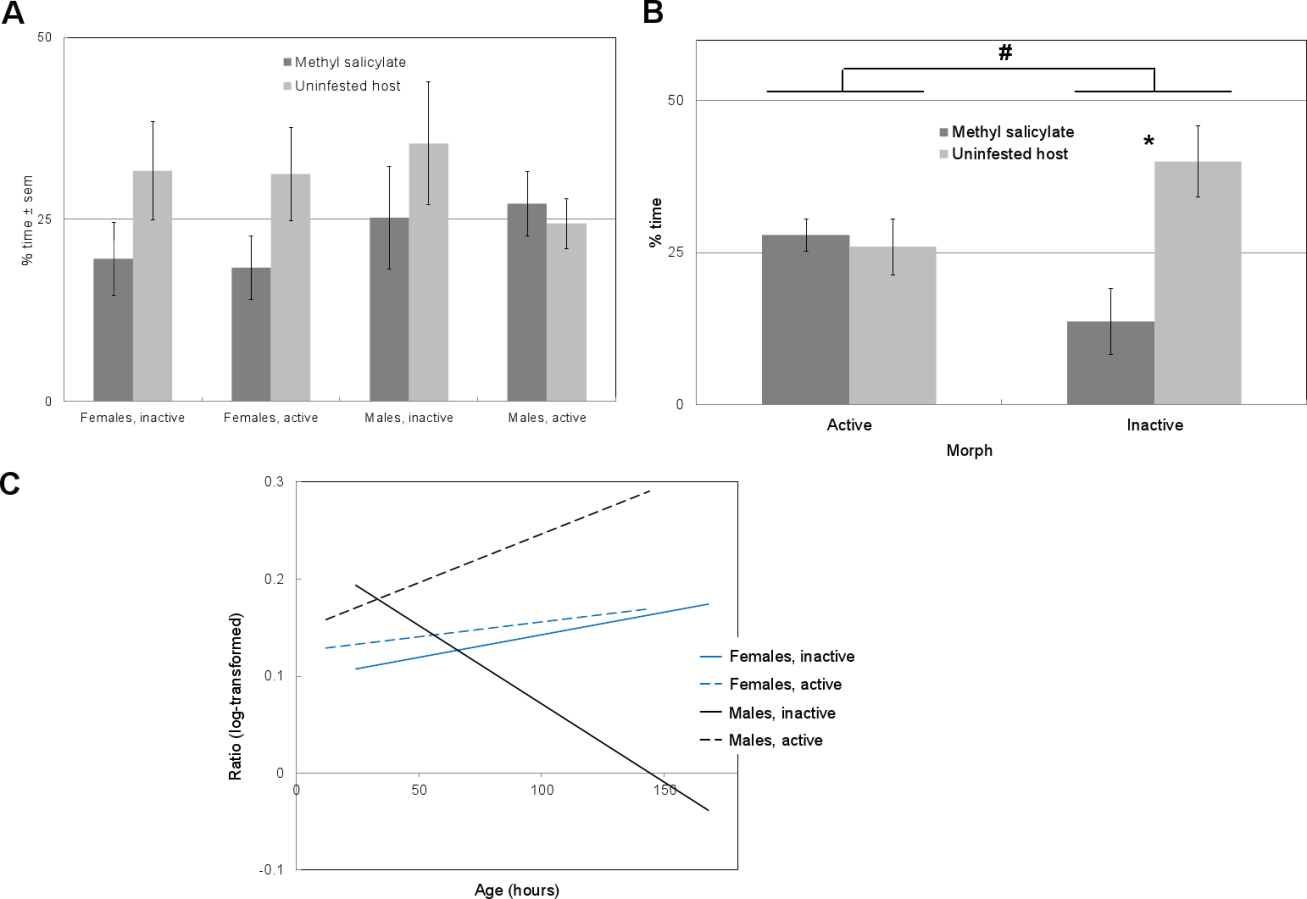
Repellency and attraction behaviour in *C. maculatus*, with low and high concentrations of methyl salicylate. a)% of time (±s.e.m.) spent in the quadrant containing methyl salicylate (regardless of concentration), and in the quadrant containing uninfested cowpea; b) repellency of methyl salicylate (±s.e.m.) by flight form at 1 mg/ml; c) graph showing role of age, sex and morph in repellency of 1 mg/ml methyl salicylate (expressed as log-transformed ratio of time spent in methyl salicylate quadrant over time spent in any treatment quadrant), in which repellency is shown to increase with age in inactive males. Methyl salicylate was overall significantly repellent for females and inactive morph insects, but was not significantly repellent for males and active morph insects. 25% would indicate no preference for a quadrant in a) and b). Dots and stars indicate a difference between bars or groups with a significance threshold of: **#**  =  0.1, *  =  0.05, **  =  0.01.

The quadrant containing methyl salicylate odour was visited for only 22.3±2.6% of their time by beetles on average (Fig. 3A), less than the host odour quadrant (31.7±3.3%). However, this narrowly falls short of the significance threshold and so methyl salicylate cannot be said to have volatile repellence for *all C. maculatus* adults based on this evidence alone (*t*-test, *t* = 1.58, *df* = 87, *p* = 0.059).

However, at the higher concentration (1 mg/ml) differences between different adults of *C. maculatus* emerged (Fig. 3B), and for some subgroups of insects, significant repellence was apparent, showing a trend for inactive adults to be more repelled by methyl salicylate than active ones (GLM, *F* = 3.78, *df* = 27, *p* = 0.065). More importantly, there were interactive effects between age and flight form (GLM, *F* = 5.51, *df* = 23, *p* = 0.029) and age, sex and flight form (GLM, *F* = 8.51, *df* = 21, *p* = 0.0082), showing that inactive males in particular become more strongly repelled by methyl salicylate with age, whilst females show a more consistent repellence profile and active males tend to become less strongly repelled by methyl salicylate with age (Fig. 3C).

The data thus show that the response of adult *C. maculatus* to a repellent compound is mediated in part by interactive effects of age, sex and flight morph, rather than varying randomly between individuals or being expressed consistently by all individuals. The results show that although not effective against all individuals of the species, methyl salicylate may have potential against some particular types of individual (specifically older beetles of the inactive morph, in particular females – which is the group most likely to oviposit on stored cowpea). However, had the evaluation only considered all bruchids grouped together, it may have been difficult to draw strong conclusions about the effect of the compound on their behaviour.

## Discussion

Previous studies in aphids have shown differences in behaviour between alate and apterous forms of the adults, most noticeably in reproduction (alate adults reproduce later and produce fewer offspring) [39], but also in olfactory-mediated behaviours [13]. In experiments with the cowpea aphid *Aphis craccivora*, the alate responded more strongly to the odour of cowpea, but less strongly to the odour of dolichos (*Lablab purpureus*), relative to the apterous form [13], [40]. Our study shows that analogous differences in orientation towards host material can similarly occur in different morphs of a coleopteran species. Adult morph type in *C. maculatus* interacted significantly with age to affect attraction to bruchid-infested cowpea and also to material with no infestation. In addition, we found that overall attraction to any kind of cowpea stimulus, whether it was infested or not, was stronger in the inactive morph than in the active one. As the active morph has evolved for dispersal and is actively seeking new host material on which to oviposit, this is perhaps a surprising result, but it should be noted that the host-seeking behaviour of *C. maculatus* in the field is to initially infest the beans of fresh cowpea, rather than dried cowpea in food storage facilities. Previous studies have associated the active form primarily with this behaviour, and considered the inactive form to be the primary pest of dried, stored legumes [41], [42] – although both will oviposit willingly on dried cowpea. A study by Messina [43] supports this view, showing green pods were preferred as an oviposition substrate over fully mature pods by both morphs; however, in that study beetles contacted the material so the cues involved may be more complex. Innate odour-mediated orientation towards dried legumes of any kind is expected to be higher in the inactive form, a pest of stores, relative to the active form, while the active form is likely to be preferentially attracted to volatiles from fresh cowpea.

Age- and sex-dependent differences were also observed in the preferences for cowpea. The females' increased attraction to cowpea odours overall relative to that of the males is likely to be because females locate cowpea directly, as a host material for their offspring. Males are expected to be attracted cowpea, if at all, only as a place where females may be encountered (and from which virgin females may be emerging). Our results support this view: females are more strongly attracted to cowpea odours overall. Females also show a stronger preference for the scent of infested cowpea than males, perhaps surprisingly given the considerations above, as one might anticipate that females would be deterred by the prospect of competition with their offspring for food. However, this result may be because the damage to the beans induces the release of cowpea volatiles and makes them more abundant and thus easily detectable (damaged cereals have been found to be more attractive to stored cereal pests such as *Sitophilus* spp. [44] perhaps for this reason). Females that are attracted to cowpea with only early bruchid infestation may also be favoured in evolution as it makes them more likely to oviposit on safe host material rather than tainted or inappropriate materials. This may be especially true of strains that are more tolerant of larval competition [45]. In this strain, we have yet to test this trait.

We then sought to test whether intraspecific differences in orientation towards attractive stimuli were mirrored when studying behaviour of these insects in the presence of a repellent stimulus, because this would be relevant to formulating control strategies. We carried out an analogous experiment examining *C. maculatus* responses to uninfested cowpea versus methyl salicylate, a repellent compound to several storage beetles and the major volatile in the pesticidal plant *Securidaca longepedunculata* [28], [36]. We observed that responses of beetles were not consistent, i.e. inactive-morph beetles were repelled by high concentrations of methyl salicylate, whereas active-morph ones were not. There were also significant interactive effects between age and flight morph, and age, morph and sex. This differential efficacy has implications in the development and deployment of odour based control strategies and should be considered in future research, especially as inactive females (the mostly strongly repelled by the compound) are most likely to lay large numbers of eggs, and the strongest repellent effect was found among beetles of maximum reproductive age (2 days or more, but particularly 2–5 days). This also emphasises that amalgamating data may mask important behaviours, such as significant odour preferences, that occur only in a subset of the population (e.g. females, older individuals). While overall the beetles tested in our study were not consistently repelled by methyl salicylate, when considering just females, or inactive individuals at the higher concentration treatment, significant repellence was observed.

Our findings are important particularly considering that there are few examples of *Callosobruchus* sp. being conclusively repelled by any volatile. The overwhelming majority of studies on this species assume repellence based on the outcome of experiments in which the beetle could contact the stimulus (e.g. filter paper, treated host material) and, therefore, do not separate out contact-based cues from odour-based responses [6], [10], [46], [47]. Boeke and colleagues [6], [10] reported that insects always remained on the material (treated or untreated) that they first contacted and so must have made a decision prior to this contact; however, their data indicate that the preferences between olfactometer arms were only exhibited among beetles that had made contact with material. This suggests that beetles may have made the decision after approaching stimuli very closely in order to sample odours: such behaviour affects decisions in other insects which do not normally respond to volatile stimuli [48]. This may also be a weakness of many (though not all) studies on cereal pests – the efficacy of plant-derived odours or resistance mechanisms is underexplored. This makes our finding more significant: there are volatiles that can modify the behaviour of this major pest insect in the absence of any visual or contact cues. Our study strengthens the case for the development of traps, lures and protective mechanisms targeting the behaviour of those forms of the insect which are most likely to respond. By considering which insects will be affected most by an odour, attractive or deterrent, one can formulate control strategies that are tailored to the high-risk behaviours of each form (e.g. inactive form bruchids are more likely to approach crawling, rather than flying, and thus particular treatment of ground-level edges of stores with repellent compounds is important, while traps only accessible to active form individuals should be baited with odours attractive to them rather than to the inactive form).

Further research is required into the differences in physiology and behaviour between active and inactive forms of *C. maculatus*, focusing on the extent of these differences in odour responsiveness. Many studies have already examined the conditions which cause development of one morph or the other, and the life history of the two forms [20]–[23], [25], [49], [50]. However, much is unknown that would help to formulate the most effective management strategies for these insects. The active form, being capable of significant long-range dispersal, should be the primary target for preventative control methods. However, inactive females can produce far more offspring and are more important pests within the legume stores themselves.

Our study emphasises the importance of distinguishing the responses of different morphs, ages and between males and females when studying pest insect species. This is particularly relevant for the control of crop pests. Development of pest management strategies must be tested for efficacy specifically against those subgroups most likely to cause problems, and must be applied in a targeted manner for effectiveness against those individuals with consideration of their behaviour and physiology.
